# Predictive value of troponins and simplified pulmonary embolism severity index in patients with normotensive pulmonary embolism

**DOI:** 10.1186/2049-6958-8-34

**Published:** 2013-05-28

**Authors:** Savas Ozsu, Yasin Abul, Asim Orem, Funda Oztuna, Yilmaz Bulbul, Huseyin Yaman, Tevfik Ozlu

**Affiliations:** 1 Department of Pulmonary Medicine, Karadeniz Technical University, School of Medicine, Trabzon, Turkey; 2 Biochemistry, Karadeniz Technical University, School of Medicine, Trabzon, Turkey

**Keywords:** Prognosis, Pulmonary embolism, Risk scores, Troponins

## Abstract

**Background:**

To investigate whether 2 cardiac troponins [conventional troponin-T(cTnT) and high sensitive troponin-T(hsTnT)] combined with simplified pulmonary embolism severity index (sPESI), or either test alone are useful for predicting 30-day mortality and 6 months adverse outcomes in patients with normotensive pulmonary embolism(PE).

**Methods:**

The prospective study included 121 consecutive patients with normotensive PE confirmed by computerized tomographic(CT) pulmonary angiography. The primary end point of the study was the 30-day all-cause mortality. The secondary end point included the 180-day all-cause mortality, the nonfatal symptomatic recurrent PE, or the nonfatal major bleeding.

**Results:**

Overall, 16 (13.2%) out of 121 patients died during the first month of follow up. The predefined hsTnT cutoff value of 0.014 ng/mL combined with a sPESI ≥1 'point(s) were the most significant predictor for 30-day mortality [OR: 27.6 (95% CI: 3.5–217) in the univariate analysis. Alone, sPESI ≥1 point(s) had the highest negative predictive value for both 30-day all-cause mortality and 6-months adverse outcomes,100% and 91% respectively.

**Conclusions:**

The hsTnT assay combined with the sPESI may provide better predictive information than the cTnT assay for early death of PE patients. Low sPESI (0 points) may be used for identifying the outpatient treatment for PE patients and biomarker levels seem to be unnecessary for risk stratification in these patients.

## Background

Hemodynamic parameters, including systemic pressure and heart rate, and associated comorbidities such as malignancy, heart failure, or pulmonary diseases, are important prognostic factors in patients with pulmonary embolism(PE) at hospital admission [1-3]. Several models have been used to determine the prognosis of PE [4-6]. The Pulmonary Embolism Severity Index(PESI) is one of the validated scores used on admission for estimating the 30-day mortality [7]. Currently, European Society of Cardiology(ESC) guidelines recommend a risk stratification according to the presence of hypotension/shock, right ventricular dysfunction (e.g. echocardiography, spiral computed tomography, or brain natriuretic peptide testing) or myocardial injury (e.g. cardiac troponin T or I testing) [8]. In addition, clinical scores have been used to predict adverse outcomes in acute PE regardless of imaging or biomarkers [9].

Laboratory biomarkers, particularly cardiac troponins, have been shown to identify patients with a high risk for mortality and an unfavourable prognosis during the acute phase of PE [10]. A very low amount of troponin can be detected in the blood of the general population with currently available highly sensitive assays and these assays have been reported to produce measures that relate to adverse cardiovascular outcomes [8,11,12]. Elevated troponin levels have been reported in various chronic diseases apart from acute myocardial infarctions, including diabetes and chronic renal disease [10,12].

We aimed to investigate whether risk stratification by assays of cardiac troponin levels, including conventional troponin-T(cTnT) and highly sensitive troponin-T (hsTnT) combined with the simplified PESI(sPESI) improves the prediction of 30-day short term and 6 months long term clinical outcomes for PE patients. We further aimed to determine whether a combination of these tools is capable of providing important additive prognostic knowledge and particularly whether it provides a practical method for the determination of low-risk patients more easily than either test alone.

## Methods

### Study design

Prospectively the study enrolled 121 consecutive patients with normotensive acute PE. The study was approved by the local ethical committee and written informed consent was obtained from all patients.

### Patients and settings

All diagnoses in PE patients were confirmed by contrast-enhanced computerized tomographic pulmonary angiography. The diagnosis of PE was based on the clinical probability and a positive (≥500 μg/L) D-dimer ELISA test [13,14]. D-dimer test was requested in case of low clinical probability only. The records of all patients diagnosed in our hospital were analyzed on admission. PE patients with shock or hypotension (high risk: defined by the ESC as a systolic blood pressure of 90 mmHg or a pressure drop of ≥ 40 mm Hg for 15 min if not caused by new onset arrhythmia) [8] were excluded from the study.

We determined test characteristics of the sPESI and of the two different cardiac troponin assays’ (cTnT and hsTnT) in their prognostic role for predicting the 30-day outcome (mortality, nonfatal recurrent venous thromboembolism, or nonfatal major bleeding) and 180-day mortality. The sPESI was calculated giving one point for the presence of every of the following parameters: (1) age > 80 years; (2) having a cancer; (3) history of chronic cardiac or pulmonary disease; (4) heart rate > 110 bpm; (5) systolic blood pressure 90 to 100 mm Hg; and (6) arterial oxyhemoglobin saturation <90% measured at the time of PE diagnosis [11]. Missing data were considered to be normal. Patients were divided in two groups, one at a low-risk (0 points) and the other at a high-risk (≥ 1 point[s]).

### Echocardiographic examination

All patients were examined by two-dimensional, pulse-wave Doppler echocardiography within the first 24 hours after a diagnosis of PE, using a Vivid 7 (GE Vingmed Ultrasound, Horten, Norway) with a 2.5-MHz transducer. The transthoracic echocardiography (TTE) examinations performed by an experienced echocardiographer were blinded to the results of biochemical assays.

Right ventricular dysfunction(RVD) was defined as dilatation of the right ventricle (end-diastolic diameter > 30 mm from the parasternal view or a right/left ventricular diameter ratio ≥ 1.0 from the subcostal or apical view), with hypokinesis of the right ventricular free wall (any view), or a tricuspid systolic valve > 30 mm-Hg from the apical or subcostal 4-chamber view [15]. The echocardiographic readers were blinded to the results of the patient data.

PE-related mortality was defined as death caused by right ventricular insufficiency or recurrent PE in the absence of an alternative diagnosis (for example, terminal cancer). A sudden or unexpected death was considered as a possible fatal PE in a patient.

### Study outcomes

The primary end point of the study was the adverse 30-day outcome, defined as death from any cause. Secondary end points were 1) nonfatal recurrent venous thromboembolism, 2) nonfatal major bleeding, 3) all-cause mortality within a 6-month follow-up period. The long-term (6-month) status of patients who had been discharged from the hospital was followed by an outpatient visit or by a telephone interview with the patient or his/her treating physician.

Nonfatal bleeding events were classified as major if they were overt and 1) occurred in a critical organ (e.g. intracranial, intraocular, or retroperitoneal hemorrhage), 2) were associated with a drop in the hemoglobin level of 2.0 g/dL or more, 3) required a transfusion of 2 units of blood or more [16].

Patients with symptoms or signs of recurrent PE were assessed with objective tests. Recurrent PE was diagnosed by the presence of a new intraluminal filling defect or an extension of a previous filling defect on computed tomography pulmonary angiography.

### Biochemical analysis

Venous blood samples were collected on admission. Troponin-T levels were determined by a quantitative electrochemiluminescence assay (Elecsys 2010; Roche, Mannheim, Germany, cut-off value <0.010 ng/ml) on admission. Samples for hsTroponin-T measures were immediately centrifuged, frozen and stored at -80°C. hsTroponin-T levels were defined by quantitative electrochemiluminescence immunoassays (Elecsys 2010 analyzer, Roche Diagnostics, Mannheim, Germany) with a cut-off value ≥ 0.014 ng/mL). A positive troponin test result was defined as a troponin level above the manufacturers assay threshold for the diagnosis of myocardial injury.

### Statistical analysis

The Kolmogorov-Smirnov test was used to assess a normal distribution of continuous variables. Data characterized by a normal distribution were expressed as mean values and standard deviation. Parameters without such distribution were expressed as median with the range. Student’s test (normal distribution) or Mann-Whitney’s (non-normal distribution) test was used for comparing the two groups. Discrete variables were compared using the Fisher exact test (chi-square test). Sensitivity, specificity, positive predictive value, negative predictive value and accuracy were calculated according to standard formulae. The prognostic relevance of the hsTnT or cTnT levels, and of the sPESI, with regard to 30-day outcomes was estimated by using a logistic regression analysis. Odds ratios (OR) and the corresponding 95% confidence intervals were calculated. P < 0.05 was considered statistically significant. Data were analyzed using SPSS statistical software.

## Results

The patients’ characteristics are summarized in Table 1. The median age was 70 years, ranging from 21 to 104 (25^th^–75^th^ percentile: 55-76) years, and 52 (43%) were males. The most frequent presenting symptom was dyspnea (75%), chest pain or pleuritic pain (49%), hemoptysis (16%), and syncope (14%). The risk factors for PE include immobility (25%), surgery (18%), cancer (23%), and other unspecified, more rare, causes (16%).

**Table 1:** Characteristic features of patients included in the study

**Demographic factors**	**All patients**	**Death (any cause) at 30 days**		**p**
		**No** **(n = 105)**	**Yes** **(n = 16)**	
Male sex	52	45	7	NS
%	43	43	44	
Median age (25^th^ to 75^th^ percentile)	70 (55-76)	70 (54-75)	77 (69-85)	0.003
Age > 80 years-(n) %	13	7	6	NS
	11	7	38	
**Clinical findings**				
Median pulse (25^th^ to 75^th^ percentile)	90 (80-108)	88 (80-100)	120 (89-129)	0.001
Pulse > 110 beats/min-(n) %	30	21	9	0.004
	25	20	56	
Median (25^th^ to 75^th^ percentile) SBP	120 (110-130)	120 (110-130)	110 (100-130)	NS
SBP < 100 mm Hg-(n) %	14	11	3	NS
	12	11	19	
Arterial oxyhemoglobin saturation (SaO_2_) < 90% (n=108)-(n)%	21	14	7	0.014
	19	15	44	
**Comorbidities**				
Cancer-(n) %	28	20	8	0.011
	23	19	50	
COPD-(n) %	9	8	1	NS
	7	8	6	
CHF-(n) %	19	15	4	NS
	16	14	25	
**Prognostic factors**				
sPESI≥1-(n) %	76	60	16	<0.001
	63	57	100	
cTn-T≥0.01 ng/mL -(n) %	50	37	13	0.001
	41	35	81	
hsTn-T≥0.014 ng/mL-(n) %	66	51	15	0.001
	55	49	94	

Data are presented as median (interquartile range) and % value.

CHF, congestive heart failure; COPD, chronic obstructive lung disease; NS, not significant; RVD, right ventricular dysfunction; SBP, systolic blood pressure; sPESI, simplified pulmonary embolism severity index.

The median hsTnT level was 0.016, ranging from 0.003 to 0.56 ng/mL (25^th^–75^th^ percentile, 0.005-0.037). A total of 66(55%) patients had hsTn–T levels ≥0.014. The median cTnT level was 0.01 ranging from to 0.01-0.39, (25^th^–75^th^ percentile, 0.01-0.027).

A transthoracic echocardiogram was evaluated in 113 patients (93%). Out of these, 53 (47%) were diagnosed with RVD. Out of 53 patients with RVD, 68% had hsTnT levels ≥0.014. while 57% had cTnT levels ≥0.01 (p = 0.003, and p = 0.001 respectively).

The sPESI classified 76 patients (62.8%) in the high-risk group (≥ 1 point[s]). Patients with a sPESI high risk presented with a positive cTnT level (48%, 37 pts) and a positive hsTnT level (68.4%, 52 pts) (p=0.033, and p<0.001, respectively).

### 30-day mortality

Out of the 121 study patients, 16 (13%) died within the first month after diagnosis. In six of them (38%) the cause of death was directly related to the PE episode, whereas other deaths were caused by cancer (31%; 5 out of 16 deaths), pneumonia (12.5%; 2 out of 16 deaths), and other diseases (19%, 3 out of 16 deaths).

All the 14 patients with a low sPESI had positive hsTnT levels and 13 out of them had positive cTnT levels. None of these patients had adverse events.

As shown in Table 2, alone sPESI ≥ 1 point(s) had a higher sensitivity, and a higher negative predictive value for predicting a 30-day mortality in the present study. None of the patients with hsTnT levels < 0.014 and a sPESI < 1, or with cTnT levels < 0.014 and a sPESI < 1 (n = 31, 26%) or with cTnT levels < 0.01 and sPESI <1 (n = 33, 27%) died during the study period. Overall, the risk assessment based on a positive hsTnT level (OR 12.4, 95% CI 1.5–99.3; p = 0.018) maintained its prognostic value for a 30-day mortality when adjusted for the sPESI (OR: 9.3, 95%CI 1.1–75.4; p = 0.036).

**Table 2:** sPESI and troponins prediction rule test characteristics for 30-day mortality

	**Sensitivity, % (95% CI)**	**Specificity, % (95% CI)**	**Negative predictive value, % (95% CI)**	**Positive predictive value, % (95% CI)**
sPESI ≥ 1 point(s)	100 (76-100)	43 (33-53)	100 (90-100)	21 (13-32)
hsTn-T ≥ 0.014 ng/mL	94 (68-100)	51 (42-61)	98 (89-100)	23 (14-35)
cTn-T ≥ 0.01 ng/mL	81 (54-95)	65 (55-74)	96 (87-99)	26 (15-41)
hsTnT ≥ 0.014 ng/mL+ sPESI ≥ 1 point(s)	94 (68-10)	65 (55-74)	99 (91-100)	29 (18-43)
cTnT ≥ 0.01+ sPESI ≥ 1 point(s)	81 (54-95)	77 (68-85)	96 (89-99)	35 (21-53)
RVD on echocardiography	19 (1-32)	95 (85-99)	57 (47-67)	77 (46-94)

CI, confidence interval; RVD, Right ventricula r dysfunction; sPESI, simplified Pulmonary Embolism Severity Index.

Overall, the risk assessment based on a positive cTnT level (OR 2.9, 95% CI 1.5–5.6; p = 0.002) maintained its prognostic value for a 30-day mortality when adjusted for a sPESI (OR 6.5, 95% CI 1.7–25.2 p=0.007).

### Combination model

We investigated the combination of troponins and the sPESI with regard to risk stratification of PE. Upon univariate analysis, hsTnT levels ≥14 ng/mL plus a sPESI ≥ 1 point(s), which represents the high risk PE patients was associated with a 27.6-fold (95% CI: 3.5-217.0, p = 0.002) increased risk of 30-day mortality (Table 3). However multivariate analysis of these parameters was not statistically significant (data not shown).

**Table 3:** Predictors of 30-day mortality (univariate analysis)

	**OR 95%**	**CI**	***P***
sPESI ≥ 1 point(s)	12.7	1.6-98.9	NS
RVD	5.6	1.5-21.0	0.011
hsTnT ≥ 14 pg/mL	4.0	1.4-11.2	0.009
cTnT ≥ 001	2.8	1.5-5.4	0.002
hsTnT ≥ 14 pg/mL+ sPESI ≥ 1 point(s)	27.6	3.5-217.0	0.002
cTnT ≥ 001+ sPESI ≥ 1 point(s)	14.6	3.8-55.6	<0.001

OR, odds ratio; RVD, Right ventricular dysfunction; sPESI, simplified Pulmonary Embolism Severity Index.

**Figure 1:**
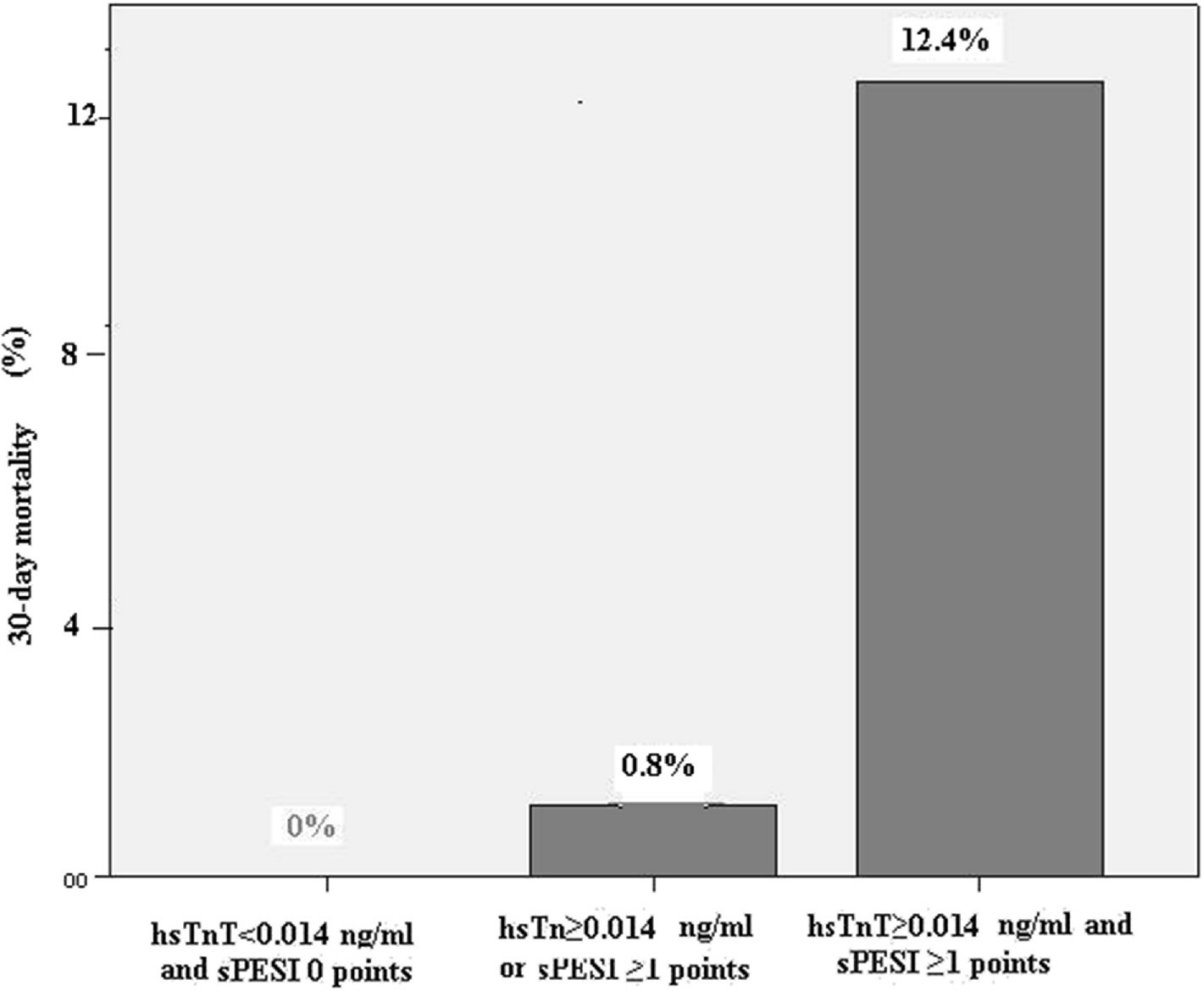
**Frequency of an adverse 30-day mortality according to baseline hsTnT levels and the sPESI.** hsTnT indicates high-sensitivity troponin T assay; sPESI, simplified PulmonaryEmbolism Severity Index.

The 30-days mortality rate rose from 0% to 0.8% in patients with sPESI ≥ 1 or hsTnT ≥ 0.014 ng/mL, and further to 12.4% in those with sPESI ≥ 1 and hsTnT ≥ 0.014 ng/mL (p < 0.001; Figure 1).

**Figure 2:**
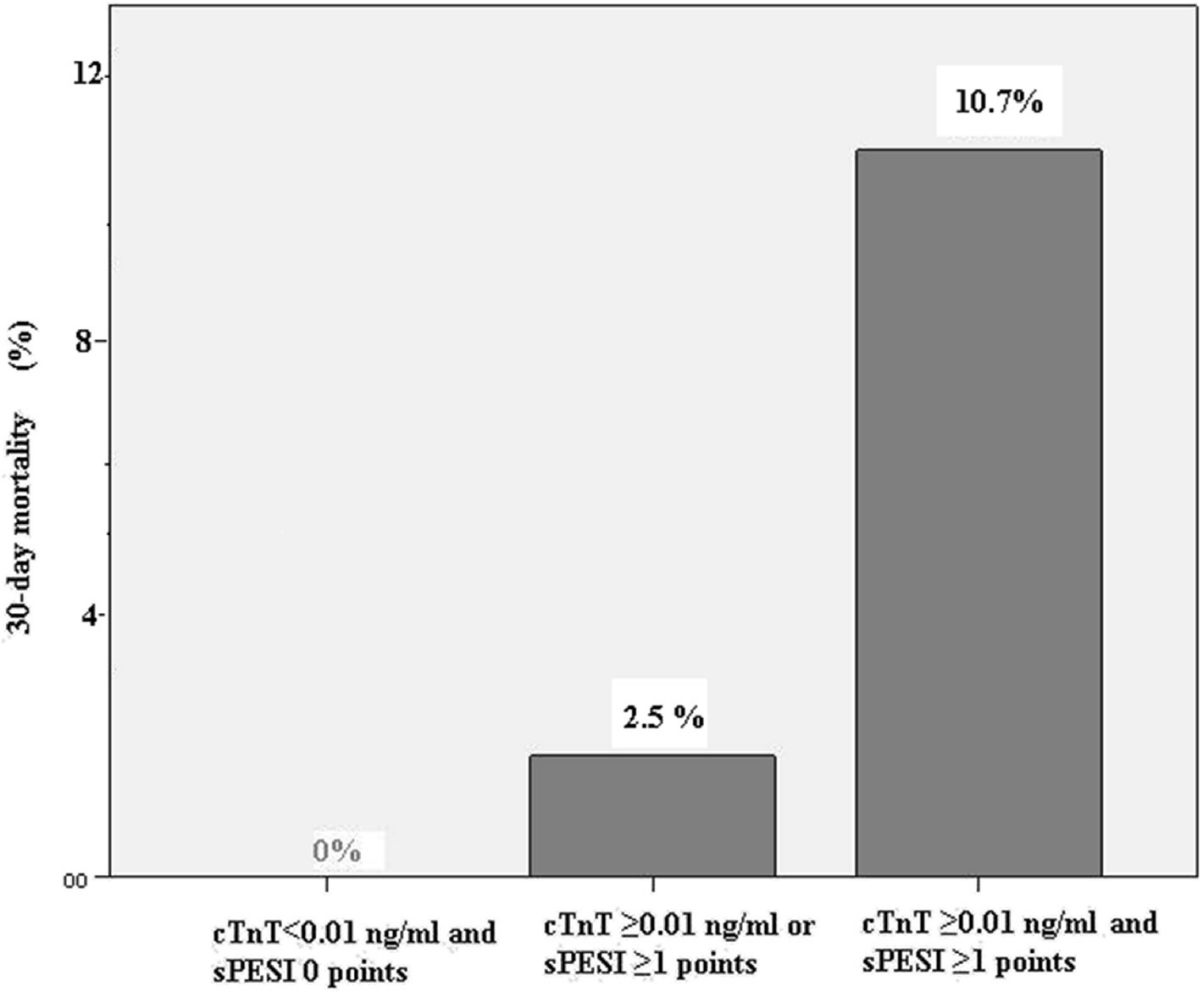
**Frequency of an adverse 30-day mortality according to baseline cTnT levels and the sPESI.** cTnT indicates conventional troponin T assay; sPESI, simplified PulmonaryEmbolism Severity Index.

The 30-days mortality rate rose from 0% to 2.5% in patients with sPESI≥1 or cTnT levels≥0.01, and further to 10.7% in those with sPESI ≥1 and hsTnT ≥0.014 ng/mL (p < 0.001; Figure 2).

### sPESI and hs-Tn-T for prediction of 6-month outcome

Overall, 28 patients (21.1%) reached the secondary end point including nonfatal recurrent venous thromboembolism (n = 3), nonfatal major bleeding (n = 3) and all-cause mortality within a 6-month period.

During the follow-up period, a total of 22 deaths (18% of the all patients) was recorded. Out of these, 16 (13%) were due to the initial PE event (all within the first 30 days), and 1 (1%) to fatal recurrent PE (only one occurred after day 30). Also, 5 deaths (4%) were due to a malignancy. Out of 55 patients with hsTnT <0.014 ng/mL on admission, 3 (5.5%) died; of 45 patients with a sPESI of 0, one (2.2%) died; of 72 patients with cTnT level <0.01 on admission, 6 (9%) died. Alone sPESI ≥ 1 point(s) has the highest negative predictive value and sensitivity for the long-term adverse outcomes (Table 4).

**Table 4:** sPESI and troponins prediction rule test characteristics for 180-day outcome

	**Sensitivity, % (95% CI)**	**Specificity, % (95% CI)**	**Negative predictive value, % (95% CI)**	**Positive predictive value, % (95% CI)**
sPESI ≥ 1 point(s)	86 (66-95)	44 (34-55)	91 (78-97)	31 (22-43)
hsTn-T ≥ 0.014 ng/mL	79 (56-91)	53 (42-63)	89 (77-95)	33 (23-46)
cTn-T ≥ 0.01 ng/mL	68 (48-83)	67 (56-76)	87 (77-94)	38 (25-53)
hsTnT ≥ 0.014 ng/mL+ sPESI ≥ 1 point(s)	75 (55-86)	67 (56-76)	90 (80-95)	40 (27-55)
cTnT ≥ 0.01+ sPESI ≥ 1 point(s)	64 (44-81)	80 (70-87)	88 (79-94)	49 (32-65)

CI, confidence interval; sPESI, simplified Pulmonary Embolism Severity Index.

## Discussion

There are three main conclusions that follow from the present results. Firstly, the sPESI is more useful for predicting the long term adverse outcomes. Secondly, a combination model of hsTnT levels ≥ 0.014 ng/mL with a sPESI ≥ 1 may be used for the short term mortality risk. Thirdly, sPESI ≥ 1 showed the high negative predictive value (100%) for 30-day mortality but PE patients had various clinical presentations leading to different prognostic and therapeutic approaches. Accurate risk stratification with precise diagnostic tools is of crucial importance.

Right ventricular dysfunction on echocardiography/computed tomography as well as cardiac markers, including troponins and clinical scores, are important tools for accurate risk stratification. There is only one study comparing the sPESI with any troponins(including hsTnT) levels, but this study population was composed of patients with massive PE [17]. Another study only compared the prognostic role of the hsTnT assay and of the sPESI [18]. Thus, comparisons of 2 cardiac troponins (hsTnT and cTnT) combined with the sPESI or alone are limited in the literature.

In our study three patients in the group with negative cTnT levels died in the early period compared to only one patient who died among the groups with negative hsTnT levels. The negative predictive value for the sPESI was 100% for a 30-day mortality. We agree with Lankeit et al. who found a 100% NPV of PE for the 30-day mortality [18]. In addition, no patient in the group with a combination of negative hsTnT levels and sPESI < 1 died. As a result it was found that, although the negative hsTnT level had a similar performance as the low sPESI for predicting adverse outcomes, it was obviously superior to the cTnT assay.

Mortality of PE has been defined to be <1% in patients without RVD on echocardiography or computerized tomography, and also with no elevations in biomarkers [9]. However, advanced age and associated comorbidities may increase the mortality for PE. Therefore, the addition of the sPESI to the biomarkers and to RVD may provide more accurate risk stratification of PE. Any patient with PE and one of the following factors (age over 80, presence of cardiopulmonary diseases and presence of cancer) has been classified as high risk patient, thus causing an overestimation of the risk stratification for these PE patients. Therefore, specificity of the high risk sPESI is lower than that of cardiac biomarkers and of right ventricular dysfunction on echocardiography for PE mortality [17,19]. The specificity of echocardiography decreases from 78% to 17–22% in PE patients with accompanying comorbidities [8]. However, a favourable prognosis of PE can be easily estimated when the sPESI is low. According to the sPESI model the 30-day overall mortality in the low risk group has been reported to be between 0% and 2% [16,17]. Fourteen patients with a positive hsTnT assay had a low sPESI in our study. However, none of these PE patients with positive ahsTnT level had an adverse event. Relatively to the good prognosis for PE patients with a low sPESI, the hsTnT assay may be an unnecessary marker, especially in those patients who are in the low sPESI group. Interestingly, in the present study the 30-day mortality was found to be 0.8 for patients that only had an hsTnT ≥ 0.014 ng/mL or a sPESI ≥ 1. Lankeit et al. found a 3.6% adverse outcome in the same patients population. However, one third of their study population had renal failure which could be associated with positive troponin values in that study [18]. Therefore combination models may provide much more information about the prognosis of PE patients and prevent unnecessary interventions in these groups of patients. Outpatient management of low risk patients with PE may improve the quality of life, and provide reductions of cost [20].

Combinations of prognostic tools including multiple biomarker assay, biomarker plus right ventricular dysfunction (both on echocardiography/tomography) and PESI (not simplified) plus shock index were found to be more predictive than biomarkers and PESI only for the risk stratification of PE patients [21-24]. In patients with hsTnT level ≥ 0.014 ng/mL plus a sPESI ≥ 1 there was a 94% risk of 30 day all-mortality and a mortality rate 12.4%. Lankeit et al. reported the 30 day adverse outcome as 10.4% in the same group [18]. In SWIVTER study it was found that patients with a high sPESI plus any positive troponin test (conventional troponin T or I, highly sensitive troponin T) had a mortality rate of 10.3% [17]. Moreover, the mortality rate was found to be 15.4% in a group where the PESI (not simplified) was combined with troponin-I in another study [25]. It remains unclear whether thrombolysis may improve the early and long-term clinical outcomes of selected normotensive patients with a high risk score and/or with biomarker positivity. The PEITHO trial, a prospective, multicenter, international, randomized, double-blind study is currently comparing thrombolysis with tenecteplasevs placebo in a normotensive patient group with confirmed PE (NCT00639743).

Of course there are some limitations in the present study. Firstly, our study population is relatively small, and secondly, we did not study hsTnT testing at the 3^rd^ hour of admission which has 100% negative predictive value for the exclusion of myocardial infarction. In addition, when interpreting our results it should also be considered that no autopsy was performed. The third concern may be related to the recurrent PE which was diagnosed by the presence of a new intraluminal filling defect or an extension of a previous filling defect on computed tomography pulmonary angiography. Because the whole diagnosis of PE patients was initially confirmed by contrast-enhanced computerized tomographic pulmonary angiography in the present study, we did not use perfusion lung scan which could be feasible for the recurrent PE and for the follow up of those PE patients.

## Conclusion

Although the present study was conducted on a limited number of patients, the hsTnT assay combined with the sPESI may provide more predictive information than the cTnT assay for the prognosis of PE. Particularly, a low sPESI may be used for the identification of outpatient treatment options and in these patients biomarker levels seem to be unnecessary for the prognosis of PE.

The study was accepted as a poster by ERS 2012 congress. This study was conducted at Farabi Hospital, Trabzon, Turkey.

## Competing interests

The authors of this manuscript have no conflicts of interest or any personal/financial support or involvement with organization(s) with financial interest in the subject matter.
